# The 2-(Triphenylsilyl)ethoxycarbonyl-(“Tpseoc”-) Group: A New Silicon-Based, Fluoride Cleavable Oxycarbonyl Protecting Group Highly Orthogonal to the Boc-, Fmoc- and Cbz-Groups

**DOI:** 10.3390/molecules16064695

**Published:** 2011-06-07

**Authors:** Martin Golkowski, Thomas Ziegler

**Affiliations:** Institute of Organic Chemistry, University of Tübingen, Auf der Morgenstelle 18, 72076 Tübingen, Germany

**Keywords:** Peterson-elimination, protecting group, silicon, fluoride ion, acid stable

## Abstract

Starting from 2-(triphenylsilyl)ethanol a new oxycarbonyl protecting group cleavable by fluoride ion induced Peterson-elimination has been developed. Known 2-(triphenylsilyl)ethanol has been prepared from commercially available triphenylvinyl-silane by a hydroboration-oxidation sequence using the sterically hindered borane reagent 9-BBN. The silyl alcohol was subsequently transformed into its chloroformate, imidazolylcarboxylic acid ester and *p*-nitrophenyl carbonate and used in standard protocols for the formation of carbamates and carbonates. The Tpseoc group proved to be highly resistant against acidic conditions applied in removal of *tert*-butyl esters and the *t-*Boc-group. It also withstood catalytic hydrogenation, treatment with morpholine, methylhydrazine and Pd-reagents/allyl-scavanger combinations, conditions required to cleave Cbz-, Fmoc-, phthalimide- and Alloc-groups. The Tpseoc-group is cleaved upon treatment with TBAF/CsF at 0 °C or r.t. with cleavage times reaching from <10 min. to 24 h. Its orthogonality, ease of cleavage and UV-detectability makes the Tpseoc-group a promising alternative to other widely used silicon based amine protecting groups like the Teoc- and SES-groups.

## 1. Introduction

Masking potentially reactive sites of a polyfunctionalized organic molecule with appropriate protecting groups is a fundamental process in modern synthetic organic chemistry. Regardless of whether the target of a synthesis is a complex secondary metabolite, a protein, oligonucleotide or saccharide, they all contain functional groups like carbonyl moieties, carboxylic acid groups, alcohols, amines, among others, which might interfere with a process that converts another functional group in the desired manner. In order to navigate through the networks of reactivity in complex organic molecules a tremendous variety of protecting groups has been developed in the past and the reports of an ongoing search for more elaborated protecting strategies fill the pages of ever growing comprehensive works on the topic [1]. The field of peptide synthesis delivers illuminating insight into protecting group chemistry, as most of the functional groups mentioned above appear in this substance class. Especially protection of the amine moiety in amino acids underwent a constant evolution from simple amides to more sophisticated carbamates finally resulting in the triumvirate of amine protecting groups consisting of the Boc- [2], Cbz- [3] and Fmoc-group [4]. Those three justify their superiority by the ease of installation, mild cleavage conditions, the excellent orthogonality among each other and not the least, the vast experience gained by the community of synthetic organic chemists since their introduction in the 1930s and 70s. During our investigation of the synthesis of glycopeptide mimetics we were prospecting for an amine protecting group which should be specifically cleavable by fluoride ions under mild conditions, but at the same time resistant enough to survive the acidic conditions applied to cleave the Boc-group. Thorough examination of the literature revealed that there are only few silicon based amine protecting groups cleavable by fluoride ions with almost none of them matching our demands. By far the most popular among them is the Teoc-group [5], based on the 2-(trimethylsilyl)ethyl-(Tmse-) moiety first described in the context of a protecting-strategy for peptide synthesis by Sieber [6]. The Teoc-group is unfortunately prone to acidolysis and is not orthogonal to the Boc-group. Another frequently used amine protecting group is the sulfonamide based SES-group [7] which in turn suffers from the necessity of somewhat harsh cleavage conditions. Two at first glance very useful protecting groups appeared to be the diphenylsilyldiethylene-(DPside-) group [8] and the triisopropylsilyloxycarbonyl-(Tsoc-) group [9]. The former is introduced into molecules bearing an amine moiety via a nucleophilic substitution reaction of bis[2-(*p*-toluenesulfonyloxy)ethyl]-diphenylsilane resulting in the formation of a 1-aza-4-silacyclohexane-derivative. Although possessing advantageous orthogonality to a variety of other amine protecting groups, the fact that it retains the basic character of the amine moiety and its limitation to sterically unhindered primary amines limit the applicability of the DPside-group. The above mentioned Tsoc-group seems so far to be the most attractive option, as it can be attached to relatively electron poor and sterically hindered primary and secondary amines. It is orthogonal to the Fmoc-, Cbz- and Boc-group and cleavage kinetics are very promising. One flaw might be that Tsoc-group can’t be attached to very electron poor amines or alcohols. In addition the procedure involved in the installation of the Tsoc-group turns out to be somewhat laborious compared to the Teoc- and SES-group, where storable activated formate reagents are used in standard protocols for their introduction. In the course of our investigations we finally stumbled across 2-(triphenylsilyl)ethanol, which was used previously as a phosphate-ester protecting group in oligonucleotide synthesis [10]. The increased electronegativity of the triphenylsilyl-moiety lead to the assumption that the silicons β-effect might be sufficiently diminished to prevent the alcohol and its derivatives from undergoing acid induced Peterson-elimination [11] as observed in Teoc-derivatives. At the same time we expected superior liability to elimination induced by attack of a nucleophilic species like fluoride or hydroxyl ions at silicon due to its increased electrophilicity. Despite the estimated favorable properties of the 2-(triphenylsilyl)ethyl moiety mentioned, we found no further evidence in the literature of its use in any kind of protecting strategy, leading to the opinion that an investigation of an amine protecting group based on the oxycarbonyl derivative of 2-(triphenylsilyl)ethanol might turn out as a promising endeavor.

## 2. Results and Discussion

**Scheme 1:**
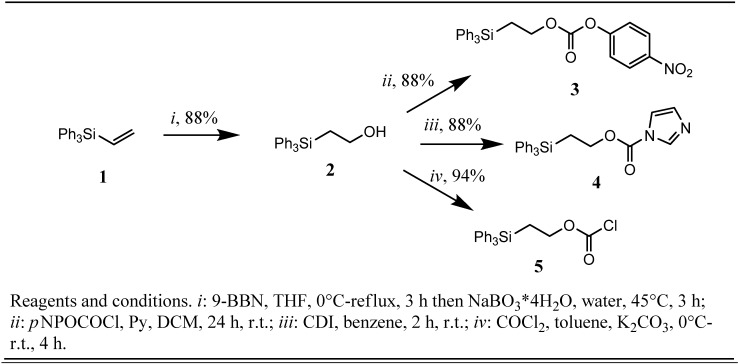
Preparation of Tpseoc-reagents.

2-(Triphenylsilyl)ethanol (**2**) was previously synthesized in only moderate yields (25-30%) by hydrosilylation of vinyl acetate with triphenylsilane employing dichlorodirhodium tetracarbonyl as the catalyst [10] or, much more efficiently, by treatment of ethylene oxide with triphenylsilyl lithium [12]. Starting from commercially available triphenylvinylsilane (**1**) we chose instead to use a straight-forward hydroboration-oxidation sequence to synthesize silyl alcohol **2** employing sterically hindered borane 9-BBN and, in regard to the expected susceptibility to elimination, the mild oxidant NaBO_3_•4H_2_O yielding alcohol **2** in an excellent yield of 88%. Borane-THF complex was also tested in the hydroboration step resulting in formation of a mixture of regioisomeric 1-(triphenylsilyl)ethanol and **2** in a ratio of 2:3 (unpublished results). This finding does not differ significantly from the regioisomer distribution observed in the hydroboration/oxidation of trimethylvinylsilane with BH_3_•THF and 9-BBN reported by Brown *et al.* [13]. Treatment of β-silyl alcohol **2** with *p*-nitrophenyl chloroformate, carbonyldiimidazole (CDI) [14] or phosgene [15], respectively resulted in a clean conversion of **2** to the corresponding *p*-nitrophenyl-2-(triphenylsilyl)ethyl carbonate (**3**),1*H*-imidazole-1-carboxylic acid 2-(triphenylsilyl)ethyl ester (**4**) and 2-(triphenylsilyl)ethyl chloroformate (**5**) in yields of 88%, 88% and 94%, respectively (Scheme 1). 

**Scheme 2:**
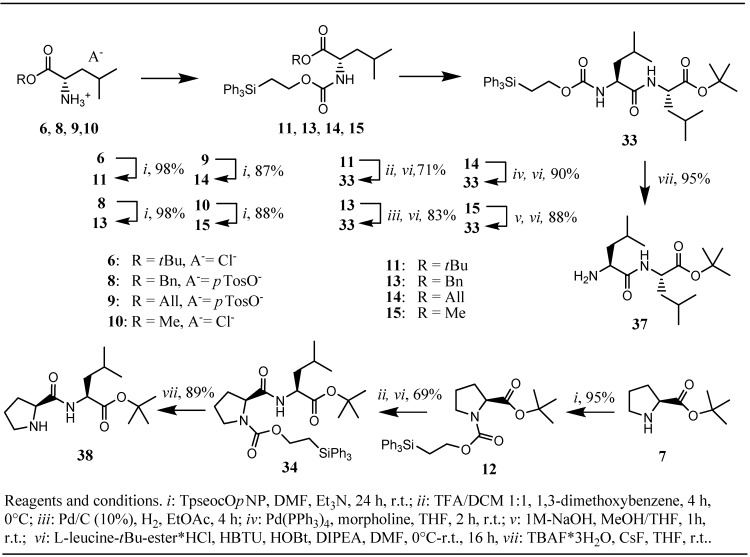
Synthesis and testing of Tpseoc-derivatives of L-leucine- and L-proline esters.

**Scheme 3:**
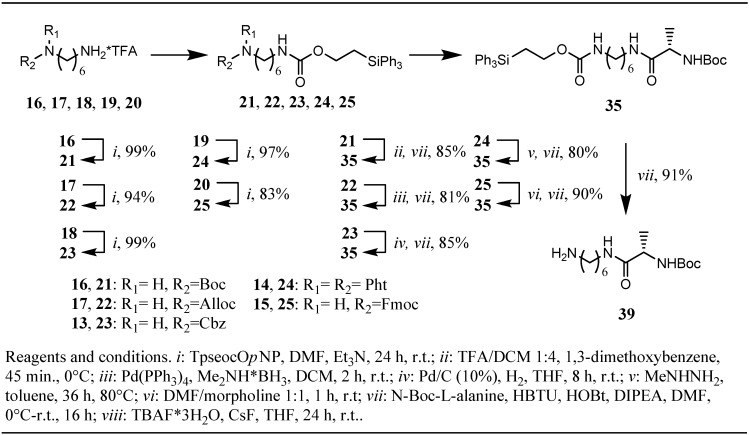
Synthesis and testing of Tpseoc-protected diamines.

The two former compounds **3** and **4** are crystalline, shelf stable solids and showed no sign of decomposition, as indicated by TLC, even after several weeks of storage at room temperature. Chloroformate **5** was obtained as crystalline solid by crystallization from dry *n*-hexane and retained its reactivity over a period of at least two months of storage in the refrigerator at −20 °C under an atmosphere of nitrogen. With the appropriate tools in hand the next step in our investigations was planned to be the Tpseoc-protection of a series of different L-leucine esters and L-proline *tert*-butyl ester, derivatives **11-15** (Scheme 2), and a series of variably protected 1,6-diaminohexane-derivatives, compounds **21-25** (Scheme 3). The Tpseoc-derivatives were synthesized according to general procedure **A** by reacting the corresponding ammonium derivatives of the amino acids **6-10** (Scheme 2) and diamines **16-20** (Scheme 3) with *p*-nitrophenyl-2-(triphenylsilyl)ethyl carbonate **3** in presence of triethylamin and DMF as solvent for 24 h at room temperature. 

The yields of Tpseoc-protected amino acid esters **11-15** and bisprotected diamines **21-24** obtained by this method were in the range of 99-87% (Table 1). Due to its base-sensitive nature, the Fmoc-protected diamine **25** was synthesized by treatment of the TFA-salt of Fmoc-1,6-diaminohexane **20** with chloroformate **5** and Hünig`s base in DCM for 3 h in a yield of 83%. On the next stage of our investigations, compounds **11-15** and **21-25** were designated to be tested for the stability of the Tpseoc-group under conditions necessary to cleave the ester function or the second amino protecting group in a competitive manner. First the Tpseoc-protected leucine and proline *tert*-butyl esters **11** and **12** were treated with 50% TFA in DCM, conditions usually applied to cleave *tert*-butyl esters [1], in the presence of 1,3-dimethoxybenzene as a cation-scavanger [16]. 

**Table 1:** Yields of protection and cleavage steps.

Substrate	Tpseoc-protection	Competitive cleavage/coupling	Tpseoc-cleavageTBAF/CsF in THF
H-Leu-O*t*Bu•HCl **6**	**11** (98%)	**33** (71%)	**37** (95%) *^c^*
H-Pro-O*t*Bu **7**	**12** (95%)	**34** (69%)	**38** (89%) *^d^*
H-Leu-OBn•TosOH **8**	**13** (98%)	**33** (83%)	-
H-Leu-OAll•TosOH **9**	**14** (87%) *^a^*	**33** (90%)	-
H-Leu-OMe•HCl **10**	**15** (88%) *^a^*	**33** (88%)	-
BocNH(CH_2_)_6_NH_2_•TFA **16**	**21** (99%)	**35** (85%)	**39** (91%) *^e^*
AllocNH(CH_2_)_6_NH_2_•TFA **17**	**22** (94%)	**35** (81%)	-
CbzNH(CH_2_)_6_NH_2_•TFA **18**	**23** (99%)	**35** (85%)	-
PhtN(CH_2_)_6_NH_2_•TFA **19**	**24** (97%)	**35** (80%)	-
FmocNH(CH_2_)_6_NH_2_•TFA **20**	**25** (83%)	**35** (90%)	-
Prasterone **26**	**27** (66%)	**-**	Prasterone **26** (88%) *^f^*
Boc-Trp(H)-OMe **28**	**29** (91%)	**-**	Boc-Trp(H)-OMe **28** (91%) *^f^*
Phe **30**	**31** (86%) *^b^*	**32** (98%)	**36** (92%) *^c^*

*^a^*: purified by crystallization; *^b^*: crude yield; *^c^*: 6 h, r.t.; *^d^*: 1.5 h, r.t.; *^e^*: 24 h, r.t.; *^f^*: 10 min., 0 °C.

In both cases TLC indicated complete consumption of the starting material after 4 h at 0 °C with obviously no significant formation of side products. When we tried to obtain an analytical sample of the amorphous free acid of Tpseoc-protected L-leucine by silica gel column chromatography eluting the product with a *n*-hexane/ethyl acetate mixture containing 1% acetic acid or formic acid, we observed formation of a non-polar side product after passing the material through the column, as indicated by TLC. Changing the column material to acidic or neutral alumina with the same eluent or RP-8 silica gel eluting the free acid with a methanol/water mixture didn’t change the outcome of the purification step. Since in all other cases of ester cleavage in **11-15** and in the competetive deprotection of the amino protecting groups in **21-25** similar problems with the purification of the free acids and amines were encountered, we chose to couple the crude products of the deprotection step in a standard peptide coupling protocol with either L-leucine *tert*-butyl ester for free acids or *N*-Boc-L-alanine for free amines. By comparing the yields of the resulting peptides **33**, **34** and **35** we expected to achieve an indirect but nonetheless authentic feedback of the stability of the Tpseoc-group under the cleavage conditions examined. For the coupling step we chose to employ HBTU [17] as coupling agent, since it allows performing the coupling step in a one-pot manner and reliably leads to very high yields. 

When this strategy was applied for *tert*-butyl esters **11** and **12** the dipeptides **33** and **34** were obtained in yields of 71% and 69% respectively. In contrast, benzyl ester derivative **13**, deprotected by hydrogenation with Pd/C (10%) in ethyl acetate, allyl ester derivative **14**, cleaved by treatment with Pd(PPh_3_)_4_/morpholine in THF [18], and methyl ester derivative **15**, saponified with 1M-NaOH in THF/MeOH, yielded the same dipeptide **33** in 83%, 90% and 88% respectively. The yields obtained, together with the observation that methyl ester **15** decomposed slowly when treated with DCM/TFA 1:1 at room temperature, as indicated by TLC (half-life ca. 12 h, unpublished results), lead to the conclusion that the Tpseoc-group could very well be termed orthogonal to *tert*-butyl esters and the *t-*Boc-group, but exhibits limited stability under prolonged exposure to strong acid. Noteworthy seems the outcome of methyl ester cleavage with 1M-NaOH in ester **15** which proceeded very cleanly, as judged by TLC analysis, and lead to high yields of dipeptide **33**, suggesting that the Tpseoc-group is not as prone to hydroxyl-ion induced elimination as initially expected [10]. Examination of the competitive cleavage of a second amino-protecting group in mono Tpseoc-protected diamines **21-25** (Scheme 3) drew a quite similar picture. When *N*-Tpseoc-*N*´-Boc-protected 1,6-diaminohexane **21** was treated with 20% TFA in DCM in presence of 1,3-dimethoxybenzene complete Boc-cleavage was observed after 45 min. at 0 °C. As expected, due to the shorter duration of the exposure to acid, the yield of 85% of peptide **35** obtained in the subsequent coupling step turned out to be significantly higher compared to *tert*-butyl ester cleavage/coupling sequence applied for **11** and **12**. Cleavage of the Alloc-group in diamino-derivative **22** was achieved by treatment with *tetrakis*(triphenylphosphine) palladium(0) in DCM and BH_3_•Me_2_NH as allyl-scavenger [19], furnishing peptide **35** after the coupling step in 81% yield. 

Cbz-derivative **23** was hydrogenated with Pd/C (10%) in dry THF as solvent instead of ethyl acetate used in deprotection of **12** (no reaction). Subsequent coupling to *N*-Boc-L-alanine yielded 85% of peptide **35**. The best results for phthalimide-cleavage in diamino-derivative **24** were obtained by using methylhydrazine in toluene under anhydrous conditions.

**Scheme 4:**
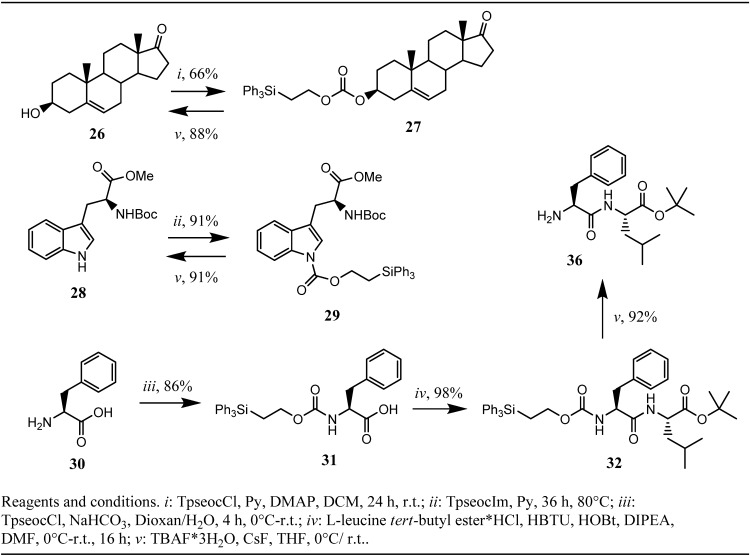
Synthesis and testing of Tpseoc-protected alcohols, electron poor amines and amino-acids.

Heating the reaction mixture to 80 °C lead to complete cleavage of the phthalimide moiety after 36 h and subsequent peptide coupling yielded 80% of peptide **35**. Treatment of Fmoc-protected diamine **25** with morpholine/DMF under anhydrous conditions followed by peptide coupling resulted in 90% recovery of peptide **35**. To further explore the scope of the Tpseoc-group we desired to protect an alcohol function as the corresponding carbonate and an electron poor amine, like the indole-nitrogen found in tryptophan. The capability of the Teoc-group as an alcohol protecting group was investigated earlier by Chattopadhyaya *et al.* and some favorable properties, especially very fast cleavage under exposure to an fluoride ion source were described [20]. In an analogical approach, prasterone **26** was transformed into its Tpseoc-carbonate with TpseocCl **5** in DCM and pyridine as the base. The reaction proceeded only slowly unless a catalytic amount of DMAP was added. After 24 h no further progression of the reaction could be observed, even upon addition of excess TpseocCl. Workup and chromatographic purification yielded moderate 66% of Tpseoc-protected prasterone **27** (Scheme 4), but the process for carbonate formation might be optimized by use of TMEDA as the base [21]. As electron poor amine *N*-Boc-L-tryptophan methyl ester **28** was chosen as a model compound and treatment with TpseocIm **4** in pyridine at 80 °C for 36 h resulted in formation of Tpseoc-protected tryptophan derivative **29** in satisfactory 91% yield (Scheme 4). Additionally chloroformate **5** was tested in a standard protection protocol widely used for installation of carbamate protecting groups in peptide chemistry, in which a free amino acid is treated with a chloroformate in water or water/dioxane (THF) in presence of a base like NaOH, carbonates or hydrogencarbonates [1]. To this end, L-phenylalanine **30** was reacted with TpseocCl **5** in water/dioxane and NaHCO_3_ as the base. Unfortunately the obtained crude Tpseoc-L-phenylalanine **31** suffered from the same problems as Tpseoc-L-leucine described above, but coupling to leucine *tert*-butyl ester using the HBTU-technique resulted in formation of dipeptide **32** in an excellent yield of 98%. (Scheme 4)

A so far unanswered question was the behavior of the Tpseoc-group under exposure to a fluoride ion source. Consequently the peptides **32**, **33**, **34** and **35** as well as the prasterone-carbonate **27** and tryptophan derivative **29** were treated with either 3 mol equivalents of TBAF•3H_2_O or a mixture of 2 mol eq. TBAF•3H_2_O/2 mol eq. CsF [8] with THF as the solvent at 0 °C or room temperature. Generally cleavage times were significantly reduced by use of TBAF•3H_2_O/CsF mixtures resulting in relatively slow Tpseoc-cleavage from the primary amine of peptide **35** (24 h, r.t.) and much faster cleavage from the primary amine of dipeptide **32** and **33** (both 6 h, r.t.) and the secondary amine in peptide **34** (90 min., r.t.). The tendency of accelerated cleavage rates going from primary amines to amines attached to sec. carbon and sec. amines might account for the release of strain induced into the molecules due to steric bulkiness of the Tpseoc-group. Both, the carbonate **27** and electron poor *N*-Tpseoc-derivative **29**, were cleaved very fast with the reaction completed after 10 minutes at 0 °C. Accordingly the observed cleavage kinetics are significantly enhanced compared to those reported for the detachment of the Teoc- and SES-group, but at the same time much slower than those described for more fluoride sensitive silicon based amino protecting groups like the Psoc-group introduced by Wagner *et al*. [22]. This suggests that the Tpseoc-group could be applied orthogonally in combination with the Psoc-group or the closely related fluoride-cleavable PTMSEL-linker, which was designed for solid-phase synthesis and possesses advantageous properties with respect to its superior fluoride sensitivity [23,24]. Concerning the occurrence of racemisation in Tpseoc-protected amino acids during the deprotection step it should be mentioned that none of the Tpseoc-derivatives deprotected according to the procedure above did show any alteration of optical rotation compared to authentic samples. Also the NMR-spectra of the free amines and alcohols lacked signals caused by isomeric products, giving rise to the assumption that under given conditions no significant racemisation of the material took place. Furthermore it should be noted that generally compounds bearing the Tpseoc-moiety exhibit a very good solubility in commonly used organic solvents and almost half of the compounds were obtained as crystalline solids making them very convenient to handle.

## 3. Experimental

### 3.1. General

Chemicals were purchased from Sigma-Aldrich (triphenylvinylsilane, HOBt, morpholine, Pd/C (10%)), Merck (DIPEA, TFA, NaBO_3_•4H_2_O), Acros Organics (9-BBN soln., Me_2_NH•BH_3_), TCI (HBTU), Fluka (phosgene soln. 20% in toluene, TBAF•3H_2_O, Pd(PPh_3_)_4_), Bachem (amino acids), Alfa Aesar (CsF) and were of the highest purity available. DCM and DMF were distilled from phosphorous pentoxide. THF, benzene, toluene and hexane were distilled from sodium/benzophenone. Pyridine, Et_3_N and DIPEA were distilled from CaH_2_. All solvents were stored over molecular sieves 3 Å under an atmosphere of nitrogen until used. NMR-spectra were recorded on either a Bruker Avance 400 or a Bruker ACX 250 spectrometer and calibrated on TMS-peak or solvent-signal peak (^1^H-CDCl_3_: 7.26 ppm; ^13^C-CDCl_3_: 77.16 ppm; ^1^H-MeOH-D_4_: 3.31 ppm; ^13^C-MeOH-D_4_: 49.00 ppm). FT-ICR-MS spectra were recorded on a Bruker Apex II FT-ICR-MS (FAB) spectrometer. Optical rotations were determined with a Perkin-Elmer Model 341 Polarimeter, melting points with a Büchi Melting Point M-560. Elemental analysis was performed on a HEKAtech Euro EA Analyzer. L-Leucine benzyl ester hydro-*p*-tosylate (**8**) and L-leucine allyl ester hydro-*p*–tosylate (**9**) were prepared following the procedures described in [25] and [26]. N-Boc-1,6-diaminohexane (**16**) was prepared following the procedure in [27]. N-Alloc-1,6-diaminohexane•TFA (**17**), N-Cbz-1,6-diaminohexane•TFA (**18**), N-Phth-1,6-diaminohexane•TFA (**19**) and Fmoc-1,6-diaminohexane•TFA (**20**) were prepared from N-Boc-1,6-diaminohexane (**16**) via introduction of the Alloc-, Cbz-, phthaloyl- and Fmoc-groups according to standard protocols, subsequent cleavage of the Boc-group with 20% TFA/DCM and were used for the syntheses without further purification. All yields given below are isolated yields determined after purification of the product either by silica gel column chromatography or crystallization and were not optimized unless noted otherwise.

### 3.2. Preparation of the Reagents for Introduction of the Tpseoc-group

*2-(Triphenylsilyl)ethanol* (**2**) [8],[28]. A 250 mL round-bottom flask equipped with gas inlet and a stirring bar was charged with triphenylvinylsilane (**1**, 1.02 g, 3.56 mmol) dissolved in dry THF (20 mL) under an atmosphere of nitrogen. At 0 °C a 0.5 M 9-BBN soln. in THF (14.2 mL, 7.12 mmol, 2 eq.) was added dropwise to the mixture, which was afterwards refluxed for 3 h, then the solution was again cooled to 0 °C and water (15 mL) was added very carefully (gas evolution!), followed by NaBO_3_•4H_2_O (3.29 g, 21.4 mmol, 6 eq.). During oxidation the temperature rose slightly to ca. 45 °C and was kept at this temperature until TLC showed complete oxidation of the borane (3 h). The mixture was then diluted with Et_2_O (200 mL) and transferred to a separatory funnel. The aqueous layer was separated and the organic layer washed twice with sat. NaHCO_3_ soln., once with brine, dried over Na_2_SO_4_ and the solvent evaporated under vacuum. Column chromatography on silica gel with the eluent mixture light petroleum ether/ethyl acetate 7:3 yielded pure title compound **2** (953 mg, 3.13 mmol, 88%) as a colorless crystalline solid. An analytical sample of alcohol **2** was obtained by crystallization from cyclohexane/*n*-hexane. m.p.: 98.5-99.5 °C (lit. [13]: 97-98.5 °C). ^1^H-NMR (400 MHz, CDCl_3_): δ 7.58-7.53 (m, 6H, aryl-Si), 7.47-7.36 (m, 9H, aryl-Si), 3.93-3.86 (m, 2H, C**H**_2_OH), 1.89-1.83 (m, 2H, SiC**H**_2_), 1.46 (t, *J* = 4.4Hz, 1H, O**H**). ^13^C-NMR (100.6 MHz, CDCl_3_): δ 135.7 (aryl, *meta*), 134.5 (aryl, *ipso*), 129.8 (aryl, *para*), 128.1 (aryl, *ortho*), 59.9 (**C**H_2_OH), 18.9 (Si**C**H_2_).

*4-Nitrophenyl-2-(triphenylsilyl)ethyl carbonate* (**3**). In a 100 mL round-bottom flask equipped with gas inlet and a stirring bar silyl alcohol **2** (1.95 g, 6.41 mmol) was dissolved in dry DCM (30 mL) under a nitrogen atmosphere. The solution was cooled to 0 °C, pyridine (2.6 mL, 32.05 mmol, 5 eq.) and 4-nitrophenyl chloroformate (1.61 g, 8.01 mmol, 1.25 eq.) were added and the mixture stirred for 24 h at r.t.. Thereafter the solution was transferred to a separatory funnel, diluted with DCM (150 mL), washed twice with 1M-NaHSO_4_ soln., once each with sat. NaHCO_3_ soln. and brine, dried over Na_2_SO_4_ and the solvent removed under vacuum. The residual viscous oil was subjected to silica gel column chromatography with the eluent mixture *n*-hexane/ethyl acetate 4:1 yielding mixed carbonate **3** (2.63 g, 5.61 mmol, 88%) as a pale yellow crystalline solid. An analytical sample was obtained by crystallization from cyclohexane/*n*-hexane. m.p.: 105.5-106 °C. FT-ICR-MS: m/z [M+Na]^+^ calcd for C_27_H_23_NO_5_SiNa: 492.1238, found: 492.1234. Anal. calcd for C_27_H_23_NO_5_Si: N, 2.98; C, 69.06; H, 4.94; found: N, 2.88; C, 68.98; H, 4.95. ^1^H-NMR (400 MHz, CDCl_3_): δ 8.30-8.24 (m, 2H, aryl-*p*NP), 7.61-7.54 (m, 6H, aryl-Si), 7.5-7.38 (m, 9H, aryl-Si), 7.36-7.3 (m, 2H, aryl- *p*NP), 4.54-4.48 (m, 2H, C**H**_2_OH), 2.09-2.02 (m, 2H, SiC**H**_2_); ^13^C-NMR (100.6 MHz, CDCl_3_): δ 155.7, 152.5, 145.4, 135.6, 133.5, 130.1, 128.3, 125.4, 121.9, 67.8, 14.9.

*1*H*-Imidazole-1-carboxylic acid 2-(triphenylsilyl)ethyl ester* (**4**) [15]. In a 25 mL round-bottom flask equipped with gas inlet and a stirring bar alcohol **2** (1.0 g, 3.28 mmol) was dissolved in dry benzene (6 mL) under a nitrogen atmosphere. Then CDI (639 mg, 3.94 mmol, 1.2 eq.) was added and the mixture stirred for 2 h at rt. Thereafter the solution was diluted with DCM (60 mL), transferred to a separatory funnel, washed twice with 1M-NaHSO_4_ soln., once each with sat. NaHCO_3_ soln. and brine. After evaporation of the solvent under vacuum a white solid was obtained, which was crystallized from cyclohexane/*n*-hexane to yield imidazolide **4** (1.16 g, 2.9 mmol, 88%) as colorless platelets. m.p.: 91.5-93.5 °C. FT-ICR-MS: m/z [M+Na]^+^ calcd for C_24_H_22_N_2_O_2_SiNa: 421.1343, found: 421.1342. Anal. calcd for C_24_H_22_N_2_O_2_Si: N, 7.03; C, 72.33; H, 5.56; found: N, 6.67; C, 72.44; H, 5.68. ^1^H-NMR (400 MHz, CDCl_3_): δ 7.92 (s, 1H, Im), 7.60-7.54 (m, 6H, aryl-Si), 7.49-7.37 (m, 9H, aryl-Si), 7.28-7.24 (m, 1H, Im), 7.03-7.00 (m, 1H, Im), 4.68-4.61 (m, 2H, C**H**_2_OH), 2.08-2.02 (m, 2H, SiC**H**_2_); ^13^C-NMR (100.6 MHz, CDCl_3_): δ 148.7, 137.1 (Im), 135.5, 133.5, 130.5 (Im), 130.1, 128.3, 117.1 (Im), 66.7, 14.6.

*2-(Triphenylsilyl)ethyl chloroformate* (**5**) [16]. In a 25 mL round-bottom flask equipped with gas inlet and a stirring bar silyl alcohol **2** (1 g, 3.28 mmol) was dissolved in dry toluene (5 mL) under a nitrogen atmosphere. After addition of freshly dried K_2_CO_3_ (453 mg, 3.28 mmol, 1 eq.) the solution was cooled to 0 °C and a 20% phosgene soln. in toluene (2.42 mL, 4.59 mmol, 1.4 eq.) added dropwise over a period of 20 min. After complete addition the mixture was stirred for an additional 4 h at r.t.. Excess phosgene was then blown off in a stream of nitrogen, the residual toluene solution filtered and the solvent evaporated under vacuum. The raw chloroformate solidified after some time under vacuum and was crystallized from dry *n*-hexane to yield pure title compound **5** (1.13 g, 3.08 mmol, 94%) as colorless needles. m.p.: 79.5-80.0 °C; Anal. calcd for C_21_H_19_ClO_2_Si: C, 68.74; H, 5.22; found: C, 68.75; H, 5.29. ^1^H-NMR (400 MHz, CDCl_3_): δ 7.57-7.51 (m, 6H, aryl-Si), 7.49-7.37 (m, 9H, aryl-Si), 4.55-4.48 (m, 2H, C**H**_2_OH), 2.05-1.98 (m, 2H, SiC**H**_2_). ^13^C-NMR (100.6 MHz, CDCl_3_): δ 150.6, 135.6, 133.3, 130.2, 128.4, 70.8, 14.8.

### 3.3. General Procedure for the Tpseoc-protection of Aliphatic Primary and Secondary Amines **A**

*General Procedure* **A**: In a 25 mL round-bottom flask equipped with gas inlet and a stirring bar mixed carbonate **3** (400 mg, 0.85 mmol) was dissolved in dry DMF (5 mL) under a nitrogen atmosphere. To the solution was added the corresponding amino acid ester ammonium derivative (0.94 mmol, 1.1 eq.) and Et_3_N (356 μl, 2.56 mmol, 3 eq.) and the resulting shiny yellow solution stirred for 24 h at r.t.. After completion of the reaction (TLC) the mixture was diluted with ethyl acetate (100 mL) and transferred to a separatory funnel, washed twice each with water and 1M-NaHSO_4_-soln., three times with 5%-Na_2_CO_3_-soln. and once with brine. The organic layer was dried over Na_2_SO_4_ and the solvent removed in vacuum. The residual crude Tpseoc-protected amino acid was then purified either by silica gel column chromatography or crystallization.

*N-2-(Triphenylsilyl)ethoxycarbonyl-L-leucine tert-butyl ester* (**11**). The protected leucine derivative **11** was prepared following the general procedure **A** from L-leucine *tert*-butyl ester (210 mg, 0.94 mmol, 1.1 eq.). The crude product was subjected to silica gel column chromatography with the eluent mixture *n*-hexane/ethyl acetate 8.5:1.5 yielding the protected amino acid **11** (430 mg, 0.83 mmol, 98%) as colorless gum. RF: 0.42 (*n*-hexane/EA 4:1). [α]_D_^20^ = −7.1° (c = 1.0, CHCl_3_). FT-ICR-MS: m/z [M+Na]^+^ calcd for C_31_H_39_NO_4_SiNa: 540.2541, found: 540.2542. Anal. calcd for C_31_H_39_NO_4_Si: N, 2.71; C, 71.92; H, 7.59; found: N, 2.77; C, 71.75; H, 8.04. ^1^H-NMR (400 MHz, CDCl_3_): δ 7.65-7.55 (m, 6H, aryl-Si), 7.49-7.36 (m, 9H, aryl-Si), 5.16 (d, 1H, *J* = 9.1 Hz, N**H**), 4.41-4.26 (m, 3H, C**H**_2_O/α-C**H**), 2.0-1.9 (m, 2H, SiC**H**_2_), 1.81-1.69 (m, 1H, γ-C**H**), 1.69-1.58 (m, 1H, β-C**H**_2_), 1.57-1.47 (m, 1H, β-C**H**_2_), 1.52 (s, 9H, *t-*Bu), 1.05-0.95 (m, 6H, δ-C**H**_3_). ^13^C-NMR (100.6 MHz, CDCl_3_): δ 172.5, 156.1, 135.5, 134.1, 129.7, 128.0, 81.6 (*t*Bu), 62.7 (**C**H_2_O), 52.9 (α-**C**H), 42.0 (β-**C**H_2_), 28.0 (*t*Bu), 24.8, 22.8, 22.0, 14.8.

*N-2-(Triphenylsilyl)ethoxycarbonyl-L-proline tert-butyl ester* (**12**). The protected proline derivative **12** was prepared following general procedure **A** from L-proline *tert*-butyl ester (160 mg, 0.94 mmol, 1.1 eq.). The crude product was purified by silca gel column chromatography with the eluent mixture *n*-hexane/ethyl acetate 4:1 yielding protected amino acid **12** (405 mg, 0.81 mmol, 95%) as a colorless gum. RF: 0.36 (*n*-hexane/EA 4:1). [α]_D_^20^ = −27.5° (c = 1.0, CHCl_3_). FT-ICR-MS: m/z [M+Na]^+^ calcd for C_30_H_35_NO_4_SiNa: 524.2228, found: 524.2225. Anal. calcd for C_30_H_35_NO_4_Si: N, 2.79; C, 71.82; H, 7.03; found: N, 2.86; C, 71.71; H, 7.42. ^1^H-NMR from the mixture of isomers (400 MHz, CDCl_3_): δ 7.60-7.54 (m, 6H, aryl-Si), 7.46-7.34 (m, 9H, aryl-Si), 4.50-4.21/4.0-3.94 (m, 3H, C**H**_2_O/α-C**H**), 3.63-3.55/ 3.51-3.43/3.41-3.34 (m, 2H, δ-**C**H_2_N), 2.19-1.71 (m, 6H, SiC**H**_2_-/β-C**H**_2_/ γ-C**H**_2_), 1.49/1.48 (s, 9H, *t-*Bu). ^13^C-NMR of the mixture of isomers (100.6 MHz, CDCl_3_): δ 172.0/171.9, 154.9/154.6, 135.5/135.5, 134.2/134.1, 129.6/129.6, 128.0/128.0, 81.0/80.9 (*t-*Bu), 62.7 (**C**H_2_O), 59.7/59.4 (α-**C**H), 46.6/46.1 (γ-**C**H_2_), 30.8/29.8 (β-**C**H_2_), 28.0/27.9 (*t-*Bu), 24.1/23.3, 15/14.9.

*N-2-(Triphenylsilyl)ethoxycarbonyl-L-leucine benzyl ester (***13***).* The protected leucine derivative **13** was prepared following general procedure **A** from L-leucine benzyl ester hydro-*p*-tosylate [25] (369 mg, 0.94 mmol, 1.1 eq.). The crude product was purified by silca gel column chromatography with the eluent mixture *n*-hexane/ethyl acetate 8.25:1.75 yielding protected amino acid **13** (456 mg, 0.83 mmol, 98%) as a colorless gum. RF: 0.4 (n-hexane/EA 4:1). [α]_D_^20^ = −7.7° (c = 1.0, CHCl_3_). FT-ICR-MS: m/z [M+Na]^+^ calcd for C_34_H_37_NO_4_SiNa: 574.2384, found: 574.23799. Anal. calcd for C_34_H_37_NO_4_Si: N, 2.54; C, 74.01; H, 6.76; found: N, 2.57; C, 74.14; H, 7.09. ^1^H-NMR (400 MHz, CDCl_3_): δ 7.57-7.51 (m, 6H, aryl-Si), 7.46-7.29 (m, 14H, aryl-Si/Bn), 5.21-5.12 (m, 2H, Bn-C**H**_2_), 4.89 (d, 1H, *J* = 8.6 Hz, N**H**), 4.43-4.35 (m, 3H, SiCH_2_C**H**_2_O/α-C**H**), 1.90-1.83 (m, 2H, SiC**H**_2_CH_2_O), 1.70-1.56 (m, 2H, β-C**H**_2_), 1.53-1.44 (m, 1H, γ-C**H**), 0.96-0.89 (m, 6H, δ-C**H**_3_); ^13^C-NMR (101 MHz, CDCl_3_): δ 172.5, 156.1, 135.7, 135.5, 134.2, 129.8, 128.7, 128.5, 128.3, 128.1, 67.1 (Bn-**C**H**H**_2_), 63.0 (SiCH_2_**C**H_2_O), 52.5 (α-**C**H), 41.8 (β-**C**H_2_), 24.8, 22.9, 21.9, 21.2, 14.8.

*N-2-(Triphenylsilyl)ethoxycarbonyl-L-leucine allyl ester* (**14**). The protected leucine derivative **14** was prepared following the general procedure **A** from L-leucine allyl ester hydro-*p*-tosylate [26] (322 mg, 0.94 mmol, 1.1 eq.). The crude product was purified by crystallization from cyclohexane/*n*-hexane yielding protected amino acid **14** (372 mg 0.74 mmol, 87%) as colorless needles. RF: 0.42 (n-hexane/EA 4:1). MP: 95-96 °C. [α]_D_^20^ = −10.2° (c = 1.0, CHCl_3_). FT-ICR-MS: m/z [M+Na]^+^ calcd for C_30_H_35_NO_4_SiNa: 524.2228, found: 524.2225. Anal. calcd for C_30_H_35_NO_4_Si: N, 2.79; C, 71.82; H, 7.03; found: N, 2.88; C, 71.95; H, 7.50. ^1^H-NMR (400 MHz, CDCl_3_): δ 7.57-7.52 (m, 6H, aryl-Si), 7.46-7.35 (m, 9H, aryl-Si), 5.97-5.86 (m, 1H, vinyl-C**H**), 5.37-5.23 (m, 2H, vinyl-C**H**_2_), 4.89 (d, 1H, *J* = 8.7 Hz, N**H**), 4.65-4.61 (m, 2H, allyl-C**H**_2_O), 4.41-4.26 (m, 3H, SiCH_2_C**H**_2_O/α-C**H**), 1.92-1.84 (m, 2H, SiC**H**_2_CH_2_O), 1.75-1.57 (m, 2H, β-C**H**_2_), 1.55-1.45 (m, 1H, γ-C**H**), 0.98-0.91 (m, 6H, δ-C**H**_3_). ^13^C-NMR (100.6 MHz, CDCl_3_): δ 173.0, 156.2, 135.6, 134.2, 131.8 (vinyl-**C**H), 129.8, 128.1, 118.8 (vinyl-**C**H_2_), 65.9 (allyl-**C**H_2_O), 63.0 (SiCH_2_**C**H_2_O), 52.5 (α-**C**H), 41.9 (β-**C**H_2_), 24.8, 23.0, 21.9, 14.9.

*N-2-(Triphenylsilyl)ethoxycarbonyl-L-leucine methyl ester* (**15**). The protected leucine derivative **15** was prepared following the general procedure **A** from L-leucine methyl ester hydrochloride (170 mg, 0.94 mmol, 1.1 eq.). The crude product was purified by crystallization from cyclohexane/hexane yielding protected amino acid **15** (355 mg, 0.75 mmol, 88%) as colorless needles. RF: 0.37 (n-hexane/EA 4:1). MP: 112.5-113 °C. [α]_D_^20^ = −7.7° (c = 1.0, CHCl_3_). FT-ICR-MS: m/z [M+Na]^+^ calcd for C_28_H_33_NO_4_SiNa: 498.2071, found: 492.2068. Anal. calcd for C_28_H_33_NO_4_Si: N, 2.94; C, 70.70; H, 6.99; found: N, 2.65; C, 70.93; H, 7.02. ^1^H-NMR (400 MHz, CDCl_3_): δ 7.57-7.52 (m, 6H, aryl-Si), 7.46-7.38 (m, 9H, aryl-Si), 4.89 (d, 1H, *J* = 8.7 Hz, N**H**), 4.39-4.27 (m, 3H, SiCH_2_C**H**_2_O/α-C**H**), 3.73 (s, 3H, OC**H**_3_), 1.91-1.85 (m, 2H, SiC**H**_2_CH_2_O), 1.73-1.44 (m, 3H, β-C**H**_2_/γ-C**H**), 0.98-0.91 (m, 6H, δ-C**H**_3_). ^13^C-NMR (100.6 MHz, CDCl_3_): δ 173.8, 156.2, 135.6, 134.2, 131.8, 129.8, 128.1, 63.0 (SiCH_2_**C**H_2_O), 52.4 (α-**C**H/OC**H**_3_), 41.9 (β-**C**H_2_), 24.8, 22.9, 21.9, 14.9.

*N-2-(Triphenylsilyl)ethoxycarbonyl-N`-tert-butoxycarbonyl-1,6-diaminohexane* (**21**). The 1,6-diamino-hexane derivative **21** was prepared following the general procedure **A** from *N*-Boc-1,6-diaminohexane **16** (237 mg, 0.94 mmol, 1.1 eq.). The crude product was purified by silica gel column chromatography with the eluent mixture *n*-hexane/ethyl acetate 7:3 yielding bisprotected diamine **21** (457 mg, 0.84 mmol, 99%) as colorless gum. RF: 0.35 (*n*-hexane/EA 7:3). FT-ICR-MS: m/z [M+Na]^+^ calcd for C_32_H_42_N_2_O_4_SiNa: 569.2806, found: 569.2802. Anal. calcd for C_32_H_42_N_2_O_4_Si: N, 5.12; C, 70.29; H, 7.74; found: N, 5.15; C, 70.24; H, 8.15. ^1^H-NMR (400 MHz, CDCl_3_): δ 7.57-7.56 (m, 6H, aryl-Si), 7.45-7.33 (m, 9H, aryl-Si), 4.60-4.44 (m, 2H, N**H**), 4.34-4.24 (m, 2H, SiCH_2_C**H**_2_O), 3.15-3.04 (m, 4H, NHC**H**_2_), 1.92-1.82 (m, 2H, SiC**H**_2_CH_2_O), 1.51-1.38 (m, 4H, chain-C**H**_2_), 1.45 (s, 9H, *t*Bu), 1.36-1.24 (m, 4H, chain-C**H**_2_). ^13^C-NMR (100.6 MHz, CDCl_3_): δ 156.7, 156.1, 135.6, 134.3, 129.8, 128.1, 79.2, 62.4, (SiCH_2_**C**H_2_O), 40.8/40.5 (NH**C**H_2_), 30.1, 30.0, 28.5 (*t-*Bu), 26.4, 26.4, 15.0.

*N-2-(Triphenylsilyl)ethoxycarbonyl-N`-allyloxycarbonyl-1,6-diaminohexane* (**22**). The 1,6-diamino-hexane derivative **22** was prepared following the general procedure **A** from N-Alloc-1,6-diaminohexane hydrotrifluoroacetate **17** (295 mg, 0.94 mmol, 1.1 eq.). The crude product was purified by silica gel column chromatography with the eluent mixture *n*-hexane/ethyl acetate 6.25:3.75 yielding bisprotected diamine **22** (424 mg 0.80 mmol, 94%) as colorless gum. RF: 0.20 (*n*-hexane/EA 7:3). FT-ICR-MS: m/z [M+Na]^+^ calcd for C_31_H_38_N_2_O_4_SiNa: 553.2493, found: 553.2496. Anal. calcd for C_31_H_38_N_2_O_4_Si: N, 5.28; C, 70.15; H, 7.22; found: N, 5.21; C, 69.70; H, 7.50. ^1^H-NMR (400 MHz, CDCl_3_): δ 7.57-7.51 (m, 6H, aryl-Si), 7.45-7.34 (m, 9H, aryl-Si), 5.98-5.86 (m, 1H, vinyl-C**H**), 5.34-5.18 (m, 2H, vinyl-C**H**_2_), 4.79 (s, broad, 1H, -N**H**), 4.56 (d, 2H, *J* = 4.9 Hz, allyl-C**H**_2_O), 4.48 (s, broad, 1H, N**H**), 4.33-4.25 (m, 2H, SiCH_2_C**H**_2_O), 3.21-3.04 (m, 4H, NHC**H**_2_), 1.91-1.82 (m, 2H, SiC**H**_2_CH_2_O), 1.54-1.38 (m, 4H, chain-C**H**_2_), 1.37-1.26 (m, 4H, chain-C**H**_2_); ^13^C-NMR (100.6 MHz, CDCl_3_): δ 156.7, 156.4, 135.6, 134.3, 133.1, 129.8, 128.1, 117.7, 65.5 (allyl-**C**H_2_O), 62.4 (SiCH_2_**C**H_2_O), 40.9/40.8 (NH**C**H_2_), 30.0, 26.3, 15.0.

*N-2-(Triphenylsilyl)ethoxycarbonyl-N`-benzyloxycarbonyl-1,6-diaminohexane* (**23**). The 1,6-diamino-hexane derivative **23** was prepared following the general procedure **A** from *N*-Cbz-1,6-diaminohexane hydrotrifluoroacetate **18** (341 mg 0.94 mmol, 1.1 eq.). The crude product was purified by silica gel column chromatography with the eluent mixture *n*-hexane/ethyl acetate 6.5:3.5 yielding bisprotected diamine **23** (487 mg 0.84 mmol, 98%) as colorless gum. RF: 0.23 (*n*-hexane/EA 7:3); FT-ICR-MS: m/z [M+Na]^+^ calcd for C_35_H_40_N_2_O_4_SiNa: 603.2650, found: 603.2653. Anal. calcd for C_35_H_40_N_2_O_4_Si: N, 4.82; C, 72.38; H, 6.94; found: N, 4.70; C, 71.95; H, 7.19. ^1^H-NMR (400 MHz, CDCl_3_): δ 7.58-7.52 (m, 6H, aryl-Si), 7.46-7.29 (m, 15H, aryl-Si/Bn), 5.11 (s, 2H, Bn-C**H**_2_O), 4.84 (s, broad, 1H, N**H**), 4.51 (s, broad, 1H, N**H**), 4.35-4.26 (m, 2H, SiC**H**_2_/C**H**_2_O), 3.23-3.04 (m, 4H, NHC**H**_2_), 1.92-1.82 (m, 2H, SiC**H**_2_CH_2_O), 1.55-1.38 (m, 4H, chain-C**H**_2_), 1.36-1.24 (m, 4H, chain-C**H**_2_); ^13^C-NMR (100.6 MHz, CDCl_3_): δ 156.7, 156.5, 136.7, 135.6, 134.3, 129.7, 128.6, 128.2, 128.1, 66.7 (Bn-C**H**_2_O), 62.4 (SiCH_2_**C**H_2_O), 41.0/40.8 (NH**C**H_2_), 30.0, 26.3, 15.0.

*N-2-(Triphenylsilyl)ethoxycarbonyl-N′-phthaloyl-1,6-diaminohexane* (**24**). The 1,6-diaminohexane derivative **24** was prepared following the general procedure **A** from *N*-phthaloyl-1,6-diaminohexane hydrotrifluoroacetate **19** (338 mg, 0.94 mmol, 1.1 eq.). The crude product was purified by silica gel column chromatography with the eluent mixture *n*-hexane/ethyl acetate 6.5:3.5 yielding bisprotected diamine **25** (472 mg, 0.82 mmol, 97%) as colorless gum. RF: 0.28 (*n*-hexane/EA 7:3). FT-ICR-MS: m/z [M+Na]^+^ calcd for C_35_H_36_N_2_O_4_SiNa: 599.2337, found: 599.2333. Anal: calcd for C_35_H_36_N_2_O_4_Si: N, 4.86; C, 72.89; H, 6.29; found: N, 4.76; C, 72.70; H, 6.62. ^1^H-NMR (400 MHz, CDCl_3_): δ 7.87-7.80 (m, 2H, Pht), 7.73-7.67 (m, 2H, Pht), 7.57-7.50 (m, 6H, aryl-Si), 7.44-7.33 (m, 9H, aryl-Si), 4.47 (s, broad, 1H, N**H**), 4.35-4.23 (m, 2H, SiCH_2_C**H**_2_O), 3.68 (t, 2H, *J* = 7.1 Hz, PhtNC**H**_2_), 3.14-3.05 (m, 2H, NHC**H**_2_), 1.91-1.81 (m, 2H, SiC**H**_2_CH_2_O), 1.73-1.63 (m, 2H, chain-C**H**_2_), 1.49-1.27 (m, 6H, chain-C**H**_2_). ^13^C-NMR (100.6 MHz, CDCl_3_): δ 168.6 (Pht-**C**ON), 156.6 (O**C**ONH), 135.6, 134.3, 134.0, 132.2, 129.7, 128.1, 123.3, 62.4 (SiCH_2_**C**H_2_O), 40.8 (NH**C**H_2_), 37.9, 29.9, 28.6, 26.5, 26.3, 15.0.

### 3.4. Preparation of Tpseoc-protected Amines and Alcohols with Various Methods

*N-2-(Triphenylsilyl)ethoxycarbonyl-N`-(9-fluorenyl)methoxycarbonyl-1,6-diaminohexane* (**25**). In a 25 mL round-bottom flask equipped with gas inlet and a stirring bar *N*-Fmoc-1,6-diaminohexane hydrotrifluoroacetate **20** (443 mg, 0.98 mmol) was dissolved in dry DCM (5 mL) together with Hünig`s base (380 µL, 2.18 mmol, 2.22 eq) under a nitrogen atmosphere. At 0 °C was then slowly added a solution of chloroformate **5** (400 mg, 1.09 mmol, 1.1 eq.) dissolved in dry DCM (5 mL). The mixture was stirred an additional 1 h at 0 °C and another 2 h at r.t.. After completion of the reaction the solution was diluted with diethyl ether and transferred to a separatory funnel. The organic layer was washed twice each with 1M-NaHSO_4_ soln., sat. NaHCO_3_ soln. and once with brine, dried over Na_2_SO_4_ and the solvent evaporated under vacuum. The crude product was then subjected to silica gel column chromatography with the eluent mixture toluene/acetone 9.25:0.75 yielding title compound **25** (540 mg 0.81 mmol, 83%) as colorless solid. An analytical sample of colorless crystals was obtained by crystallization from cyclohexane/chloroform. RF: 0.35 (toluene/acetone 9.25:0.75). MP: 147.5-148.0 °C. FT-ICR-MS: m/z [M+Na]^+^ calcd for C_42_H_44_N_2_O_4_SiNa: 691.2963, found: 691.2965. ^1^H-NMR (400 MHz, CDCl_3_): δ 7.78 (d, 2H, *J* = 7.4 Hz, Fmoc), 7.61 (d, 2H, *J* = 7.4 Hz, Fmoc), 7.59-7.52 (m, 6H, aryl-Si), 7.46-7.29 (m, 13H, aryl-Si/ Fmoc), 4.85 (s, broad, 1H, N**H**), 4.49 (s, broad, 1H, N**H**), 4.42 (d, 2H, *J* = 6.6 Hz, Fmoc-OC**H**_2_), 4.36-4.20 (m, 3H, SiCH_2_C**H**_2_O/Fmoc-CH_2_C**H**), 3.25-3.04 (m, 4H, NHC**H**_2_), 1.96-1.83 (m, 2H, SiC**H**_2_CH_2_O), 1.58-1.21 (m, 8H, chain-C**H**_2_). ^13^C-NMR (100.6 MHz, CDCl_3_): δ 156.7, 156.6, 144.1, 141.4, 135.6, 134.3, 129.8, 128.6, 128.1, 127.8, 127.1, 125.1, 120.1, 66.6 (Fmoc-O**C**H_2_), 62.4 (SiCH_2_**C**H_2_O), 47.4 (Fmoc-CH_2_**C**H), 40.9/40.8 (NH**C**H_2_), 30.0, 27.0, 26.3, 15.0.

*3β-[2-(Triphenylsilyl)ethoxycarbonyloxy]androst-5-en-17-one* (**27**). In a 5 mL Schlenk-tube equipped with a stirring bar prasterone **26** (143 mg, 0.50 mmol) was dissolved in dry DCM (2 mL) under a nitrogen atmosphere. To the solution of the steroid was added at 0 °C chloroformate **5** (200 mg, 0.55 mmol, 1.1 eq.) followed by pyridine (134 μL, 1.65 mmol, 3 eq.) and DMAP (6.7 mg, 50 μmol, 0.1 eq.). The resulting mixture was then stirred at 0 °C for 2 h and additional 24 h at r.t.. Thereafter the mixture was diluted with EtOAc, transferred to a separatory funnel, washed twice with 1M-NaHSO_4_ soln., once with sat. NaHCO_3_ soln., once with brine and dried over Na_2_SO_4_. After removal of the solvent in vacuum the residue was subjected to silica gel column chromatography with the eluent mixture *n*-hexane/ethyl acetate 3:1 yielding protected steroid **27** (207 mg, 0.33 mmol, 66%) as a colorless foam. RF: 0.41 (*n*-hexane/EA 3:1). [α]_D_^20^ = +1.2° (c = 1.0, CHCl_3_); FT-ICR-MS: m/z [M+Na]^+^ calcd for C_40_H_46_O_4_SiNa: 641.3058, found: 641.3063. ^1^H-NMR (400 MHz, CDCl_3_): δ 7.56-7.50 (m, 6H, aryl-Si), 7.46-7.35 (m, 9H, aryl-Si), 5.44- 5.40 (m, 1H, C6-**H**), 4.49-4.4 (m, 1H, C3-**H**) 4.36-4.29 (m, 2H, SiCH_2_C**H**_2_O), 2.52-2.30 (m, 3H, scaffold), 2.16-2.04 (m, 2H, scaffold), 1.99-1.82 (m, 6H, SiC**H**_2_CH_2_O, scaffold), 1.72-1.43 (m, 6H, scaffold), 1.34-1.24 (m, 2H, scaffold), 1.20-1.09 (m, 1H, scaffold), 1.07-1.00 (m, 1H, scaffold), 1.04 (s, 3H, C**H**_3_), 0.89 (s, 3H, C**H**_3_). ^13^C-NMR (100.6 MHz, CDCl_3_): δ 221.0 (**C**17), 154.6 (O**C**OO), 139.8 (**C**5), 135.6, 133.8, 129.9, 128.2, 122.2 (**C**6), 77.4, 65.7 (SiCH_2_**C**H_2_O), 51.8, 50.2, 47.6, 38.1, 36.9, 36.8, 36.0, 31.6, 31.5, 30.9, 27.8, 22.0, 20.5, 19.4, 14.8, 13.7.

*N-tert-Butoxycarbonyl-1-[2-(triphenylsilyl)ethoxycarbonyl]-L-tryptophan methyl ester* (**29**). In a 25 mL round-bottom flask equipped with gas inlet and a stirring bar *N*-Boc-L-tryptophan methyl ester (200 mg, 0.63 mmol) was dissolved in dry pyridine (5 mL) under a nitrogen atmosphere. After addition of TpseocIm **4** (375 mg, 0.94 mmol, 1.5 eq.) the mixture was heated to 80 °C for 36 h, diluted with ethyl acetate and transferred to a separatory funnel. The organic layer was washed twice with 1N-HCl soln., once with sat. NaHCO_3_ soln. and once with brine, dried over Na_2_SO_4_ and the solvent evaporated under vacuum. The residual crude product was subjected to silica gel column chromatography with the eluent mixture *n*-hexane/ethyl acetate 4:1 yielding title compound **29** (370 mg, 0.57 mmol, 91%) as colorless foam. RF: 0.3 (*n*-hexan/EtOAc 4:1). [α]_D_^20^ = +22.4° (c = 1.0, CHCl_3_). FT-ICR-MS: m/z [M+Na]^+^ calcd for C_38_H_40_N_2_O_6_SiNa: 671.2548, found: 671.2549. ^1^H-NMR (400 MHz, CDCl_3_): δ 8.13 (d, broad, 1H, *J* = 6.2 Hz, Trp), 7.61-7.54 (m, 6H, aryl-Si), 7.51-7.35 (m, 10H, aryl-Si/Trp), 7.31 (t, broad, 1H, *J* = 7.4 Hz, Trp), 7.24 (t, broad, 1H, *J* = 7.3 Hz, Trp), 7.19 (s, broad, 1H, Trp), 5.01 (d, 1H, *J* = 7.8 Hz, Boc-N**H**), 4.67-4.71 (m, 3H, SiCH_2_C**H**_2_O/α-C**H**), 3.68 (s, 3H, OC**H**_3_), 3.25-3.07 (m, 2H, β-C**H**_2_), 2.12-2.03 (m, 2H, SiC**H**_2_CH_2_O), 1.42/1.29 (s, broad, 9H, *t-*Bu_H_); ^13^C-NMR (100.6 MHz, CDCl_3_): δ 172.4 (**C**OO), 155.2 (O**C**ONH), 150.8, 135.6, 133.7, 130.5, 130.4, 128.3, 124.8, 123.7, 122.9, 119.0, 115.8, 115.4, 80.1 (*t-*Bu), 65.3 (SiCH_2_**C**H_2_O), 53.7 (α-**C**H), 52.5 (O**C**H_3_), 28.4 (*t-*Bu), 27.9, 14.8.

*N-2-(Triphenylsilyl)ethoxycarbonyl-L-phenylalanine* (**31**). In a 50 mL round-bottom flask equipped with a stirring bar L-phenylalanine **30** (205 mg, 1.24 mmol) and NaHCO_3_ (229 mg, 2.73 mmol, 2.2 eq.) were dissolved in water (10 mL). The mixture was cooled to 0 °C and a solution of TpseocCl **5** (500 mg, 1.36 mmol, 1.1 eq.) in dioxane (10 mL) was added dropwise. The resulting slurry was then stirred for 1 h at 0 °C and 4 h at r.t., dioxane removed under vacuum and the residual aqueous phase acidified with solid NaHSO_4_. After being transferred to a separatory funnel the aqueous phase was extracted three times with EtOAc, the combined organic phases washed once each with 1M-NaHSO_4_-soln., water and brine and dried over Na_2_SO_4_. After removal of the solvent under vacuum the title compound **31** (529 mg 1.07 mmol, 86%) was obtained as a colorless foam in high purity. The crude Tpseoc-protected free acid was used in the synthesis of **32** without further purification. FT-ICR-MS: m/z [M+Na]^+^ calcd for C_30_H_29_NO_4_SiNa: 518.1758, found: 518.1756. ^1^H-NMR (400 MHz, MeOH-D_4_): δ 7.51-7.44 (m, 6H, aryl-Si), 7.42-7.30 (m, 9H, aryl-Si), 7.26-7.07 (m, 5H, aryl-Phe), 4.39 (dd, 1H, *J* = 8.9 Hz/5 Hz, α-C**H**), 4.18-4.09 (m, 2H, SiCH_2_C**H**_2_O), 3.15 (dd, 1H, *J* = 13.9 Hz/5 Hz, β-C**H**), 2.90 (dd, 1H, *J* = 13.9 Hz/9 Hz, β-C**H**), 1.83-1.81 (m, 2H, SiC**H**_2_CH_2_O). ^13^C-NMR (100.6 MHz, MeOH-D_4_): δ 175.1 (**C**OOH), 158.4 (O**C**ONH), 138.4, 136.5, 135.3, 130.8, 130.3, 129.4, 129.1, 127.7, 63.8 (SiCH_2_**C**H_2_O), 56.5 (α-C**H**), 38.7 (β-C**H*_2_***), 15.8.

*N-2-(Triphenylsilyl)ethoxycarbonyl-L-phenylalanyl-L-leucine tert-butyl ester* (**32**): In a 25 mL round bottom flask equipped with gas inlet and a stirring bar the crude Tpseoc-protected L-phenylalanine **31** (300 mg, 0.61 mmol, 1.2 eq.) was dissolved in dry DMF (5 mL) together with leucine *tert*-butyl ester hydrochloride (113 mg, 0.50 mmol) and HOBt (103 mg, 0.76 mmol, 1.5 eq.) under a nitrogen atmosphere. After cooling the solution to 0 °C Hünig`s base (263 μL, 1.51 mmol, 3 eq.) and HBTU (288 mg, 0.76 mmol, 1.5 eq.) were added, the mixture stirred for 2 h at 0 °C and for additional 14 h at r.t.. Thereafter the mixture was diluted with EtOAc, transferred to a separatory funnel, washed twice each with 1N-NaHSO_4_-soln. and sat. NaHCO_3_-soln., once with brine and dried over Na_2_SO_4_. After removal of the solvent under vacuum the residual white solid was subjected to silica gel column chromatography with the eluent mixture *n*-hexane/ethyl acetate 7.75: 2.25 yielding the dipeptide **32** (323 mg, 0.49 mmol, 98%) as a colorless crystalline solid. An analytical sample of colorless needles was obtained by crystallization from cyclohexane/*n*-hexane. RF: 0.41 (*n*-hexane/EA 3:1). m.p.: 131-132 °C. [α]_D_^20^ = −7.4° (c = 1.0, CHCl_3_). FT-ICR-MS: m/z [M+Na]^+^ calcd for C_40_H_48_N_2_O_5_SiNa: 687.3225, found: 687.3228. Anal. calcd for C_40_H_48_N_2_O_5_Si: N, 4.21; C, 72.26; H, 7.28; found: N, 4.32; C, 72.41; H, 7.61. ^1^H-NMR (400 MHz, CDCl_3_): δ 7.55-7.49 (m, 6H, aryl-Si), 7.44-7.32 (m, 9H, aryl-Si), 7.29-7.13 (m, 5H, aryl-Phe), 6.19 (d, 1H, *J* = 7.9 Hz, CON**H**), 5.02 (s, 1H, OCON**H**), 4.46-4.34 (m, 2H, α-C**H**), 4.29-4.21 (m, 2H, SiCH_2_C**H**_2_O), 3.11-2.98 (m, 2H, β-C**H**_H2_-Phe), 1.86-1.78 (m, 2H, SiC**H**_2_CH_2_O), 1.59-1.49 (m, 2H, β-C**H**_2_-Leu), 1.48-1.37 (m, 1H, γ-C**H**), 1.42 (s, 9H, *t-*Bu), 0.92-0.84 (m, 6H, δ-C**H**_3_). ^13^C-NMR (100.6 MHz, CDCl_3_): δ 171.6, 170.5, 156.1, 136.4, 135.6, 134.1, 129.8, 129.5, 128.8, 128.2, 128.2, 127.1, 82.0 (*t-*Bu), 63.2 (SiCH_2_**C**H_2_O), 56.0, 51.5, 42.0, 38.4, 28.1 (*t-*Bu), 24.9, 22.8, 22.2, 14.8.

### 3.5. Competitive protecting group cleavage in N-Tpseoc-L-leucine esters ***11***, ***13***, ***14***, ***15***, N-Tpseoc-L-proline ester ***12*** and subsequent amide coupling with L-leucine tert-butyl ester. Competitive cleavage of the amino protecting groups in N-Tpseoc-1,6-diaminohexane derivatives ***21***, ***22***, ***23***, ***24***, and ***25*** and subsequent amide coupling with N-Boc-L-alanine

*General procedure* **B*** for the coupling of crude Tpseoc-protected proline and leucine free acids obtained from ester cleavage in* **11***,* **12***,* **13***,* **14***,* **15** *with L-leucine tert-butyl ester and of the crude N-Tpseoc-protected 1,6-diaminohexane free bases obtained from protecting group cleavage in* **21***,* **22***,* **23***,* **24*** and* **25*** with N-Boc-L-alanine:* In a 25 mL round-bottom flask equipped with gas inlet and a stirring bar the crude Tpseoc-protected amino acid/*N*-Tpseoc-protected 1,6-diaminohexane, obtained following the deprotection protocol given below, was dissolved in dry DMF (5 mL) together with leucine *tert*-butyl ester hydrochloride/*N*-Boc-L-alanine (1.25 eq.) and HOBt (1.5 eq.) under a nitrogen atmosphere. After cooling the solution to 0 °C Hünig`s base (1.5 eq./3 eq.) and HBTU (1.5 eq.) were added, the mixture stirred for 2 h at 0 °C and for additional 14 h at r.t.. Thereafter the mixture was diluted with EtOAc, transferred to a separatory funnel, washed twice each with 1M-NaHSO_4_-soln. and sat. NaHCO_3_-soln., once with brine and dried over Na_2_SO_4_. After removal of the solvent under vacuum the residual crude Tpseoc-protected peptides were subjected to silica gel column chromatography. 

*N-2-(Triphenylsilyl)ethoxycarbonyl-L-leucyl-L-leucine tert-butyl ester* (**33**). Pure *N*-Tpseoc-L-leucyl-L-leucine *tert*-butyl ester **33** was obtained by chromatography with the eluent mixture *n*-hexane/ethyl acetate 8.25:1.75 as a crystalline solid. An analytical sample was obtained by crystallization from cyclohexane/*n*-hexane. RF: 0.38 (*n*-hexane/EA 4:1). MP: 118.5 °C. [α]_D_^20^ = −24.8° (c = 1.0, CHCl_3_). FT-ICR-MS: m/z [M+Na]^+^ calcd for C_37_H_50_N_2_O_5_SiNa: 653.3381, found: 653.3383. ^1^H-NMR (400 MHz, CDCl_3_): δ 7.57-7.50 (m, 6H, aryl-Si), 7.45-7.34 (m, 9H, aryl-Si), 6.19 (d, broad, 1H, *J* = 8.1 Hz, CON**H**), 5.04 (d, broad, 1H, *J* = 7.8 Hz, OCON**H**), 4.52-4.44 (m, 1H, α-C**H**), 4.34-4.25 (m, 2H, SiCH_2_C**H**_2_O), 4.20-4.11 (m, 1H, α-C**H**), 1.93-1.83 (m, 2H, SiC**H**_2_), 1.72-1.57 (m, 4H, β-C**H**_2_), 1.56-1.38 (m, 2H, γ*-*C**H**), 1.45 (s, 9H, *t-*Bu), 0.96-0.89 (m, 6H, δ*-*C**H**_3_). ^13^C-NMR (100.6 MHz, CDCl_3_): δ 172, 171.9, 156.4, 135.6, 134.1, 129.8, 128.1, 81.9 (*t-*Bu), 63.1 (SiCH_2_**C**H_2_O), 53.4 (α-**C**H), 51.5 (α-**C**H), 41.8 (β-**C**H_2_), 41.5 (β-**C**H_2_), 28.0 (*t-*Bu), 24.9, 24.7, 23.1, 22.9, 22.2, 22.1, 14.9.

*N-2-(Triphenylsilyl)ethoxycarbonyl-L-prolyl-L-leucine tert-butyl ester* (**34**). Pure *N*-Tpseoc-L-prolyl-L-leucine *tert-*butyl ester **34** was obtained by chromatography with the eluent mixture *n*-hexane/ethyl acetate 7:3 as a colorless gum. RF: 0.47 (*n*-hexane/EA 3:2). [α]_D_^20^ = −50.8° (c = 1.0, CHCl_3_). FT-ICR-MS: m/z [M+Na]^+^ calcd for C_36_H_46_N_2_O_5_SiNa: 637.3069, found: 637.3067. ^1^H-NMR from the mixture of isomers (400 MHz, CDCl_3_): δ 7.59-7.51 (m, 6H, aryl-Si), 7.45-7.32 (m, 9H, aryl-Si), 7.05/6.39 (s, broad, 1H, CON**H**), 4.50/4.43 (s, broad, 1H, α-C**H**), 4.40-4.25/4.13-4.03 (m, 3H, SiC**H**_2_CH_2_O/α-C**H**), 3.50 (s, broad, 1H, δ-C**H**), 3.25/3.09 (s, broad, 1H, δ-C**H**), 2.36-1.74 (m, 6H, SiC**H**_2_/β-C**H**_2_), 1.70-1.32 (m, 12H, *γ*-C**H**_2_/*t-*Bu), 0.90 (d, broad, 6H, *J* = 6.2 Hz, *δ-*C**H**_3_). ^13^C-NMR from the mixture of isomers (100.6 MHz, CDCl_3_): δ 171.8, 171.4, 156.3/155.5, 135.6, 134.1, 129.1, 128.1, 81.7 (*t*-Bu), 63.4 (SiCH_2_**C**H_2_O), 60.7 (α-**C**H-Pro), 51.7/51.1 (α-**C**H-Leu), 47.3/ 46.7(β-**C**H_2_-Pro), 41.7 (β-**C**H_2_-Leu), 30.9, 28.2, 28.0 (*t-*Bu), 25.0, 24.6, 23.7, 22.9, 22.2, 14.9.

*N-2-(Triphenylsilyl)ethoxycarbonyl-N`(N-tert-butoxycarbonyl)-L-alanyl-1,6-diaminohexane* (**35**). Pure *N*-Boc-L-alanyl-*N*`-Tpseoc-1,6-diaminohexane **35** was obtained by chromatography with the eluent mixture *n*-hexane/ethyl acetate 2:3 as a colorless foam. RF: 0.34 (hexane/EA 2:3). [α]_D_^20^ = −2.6° (c = 1.0, CHCl_3_). FT-ICR-MS: m/z [M+Na]^+^ calcd for C_35_H_47_N_3_O_5_SiNa: 640.3177, found: 640.3177. ^1^H-NMR (400 MHz, CDCl_3_): δ 7.56-7.50 (m, 6H, aryl-Si), 7.44-7.32 (m, 9H, aryl-Si), 6.51 (s, broad, 1H, amide-N**H**), 5.24 (d, broad, 1H, *J* = 5.7 Hz, OCON**H**), 4.61 (s, broad, 1H, OCON**H**), 4.35-4.23 (m, 2H, SiCH_2_C**H**_2_O), 4.15-4.01 (m, 1H, α-C**H**), 3.27-3.18 (m, 2H, CONHC**H**_2_), 3.13-3.03 (m, 2H, CONHC**H**_2_), 1.91-1.82 (m, 2H, SiC**H**_2_), 1.52-1.39 (m, 4H, chain-C**H**_2_), 1.43 (s, 9H, *t-*Bu), 1.30 (d, 3H, J = 7 Hz, β-C**H**_3_), 1.32-1.25 (m, 4H, chain-C**H**_2_). ^13^C-NMR (100.6 MHz, CDCl_3_): δ 172.9 (**C**ONH), 156.7, 155.7, 135.6, 134.2, 129.7, 128.1, 80.0 (*t-*Bu), 62.4 (SiCH_2_**C**H_2_O), 50.2 (α-**C**H-Ala), 40.6, 39.3, 29.4, 28.4 (*t-*Bu), 26.3, 26.2, 18.6, 14.9. 

tert*-Butyl ester cleavage in N-2-(triphenylsilyl)ethoxycarbonyl-L-leucine* tert*-butyl ester* (**11**) [16]. In a Schlenk-tube equipped with a stirring bar protected amino acid **11** (127 mg, 0.249 mmol) was dissolved in dry DCM (1 mL) under a nitrogen atmosphere. To the solution were added 1,3-dimethoxybenzene (163 μL, 1.25 mmol, 5 eq.) and TFA (1 mL) and the mixture stirred at 0 °C until TLC showed complete cleavage of the ester (4 h). The solution was then evaporated to dryness and the residual crude Tpseoc-protected amino acid coupled with L-leucine *tert*-butyl ester following the general procedure **B** using L-leucine *tert*-butyl ester (70 mg, 0.311 mmol), HBTU (142 mg, 0.374 mmol), HOBt (51 mg, 0.374 mmol) and Hünig`s base (130 µl, 0.747 mmol, 3 eq.) yielding Tpseoc-protected dipeptide **33** (112 mg, 0.178 mmol, 71%).

*tert-Butyl ester cleavage in N-2-(triphenylsilyl)ethoxycarbonyl-L-proline tert-butyl ester* (**12**) [16]. In a Schlenk-tube equipped with a stirring bar protected amino acid **12** (150 mg, 0.299 mmol) was dissolved in dry DCM (1 mL) under a nitrogen atmosphere. To the solution were added 1,3-dimethoxybenzene (196 μL, 1.50 mmol, 5 eq.) and TFA (1 mL) and the mixture stirred at 0 °C until TLC showed complete cleavage of the ester (4 h). The solution was then evaporated to dryness and the residual crude Tpseoc-protected amino acid coupled with L-leucine *tert*-butyl ester following general procedure **B** using L-leucine *tert*-butyl ester (84 mg, 0.374 mmol), HBTU (170 mg, 0.449 mmol), HOBt (61 mg, 0.449 mmol) and Hünig`s base (156 µL, 0.897 mmol, 3 eq.) yielding Tpseoc-protected dipeptide **34** (127 mg, 0.207 mmol, 69%).

*Benzyl ester cleavage in N-2-(triphenylsilyl)ethoxycarbonyl-L-leucine benzyl ester* (**13**). In a 25 mL round-bottom flask equipped with gas inlet a stirring bar benzyl ester **13** (189 mg, 0.343 mmol) was dissolved in EtOAc (5 mL). A spatula tip of Pd/C (10%) was added and the mixture stirred under an atmosphere on hydrogen until TLC indicated complete consumption of the starting material (4 h). Then the solution was filtered through a pad of Celite and the solvent evaporated under vacuum. The residual crude Tpseoc-protected amino acid was then coupled with L-leucine *tert*-butyl ester (96 mg, 0.429 mmol) following general procedure **B** using HBTU (195 mg, 0.515 mmol), HOBt (70 mg, 0.515 mmol) and Hünig`s base (179 µL 1.03 mmol, 3 eq.) yielding of Tpseoc-protected dipeptide **33** (181 mg, 0.284 mmol, 83%).

*Allyl ester cleavage in N-2-(triphenylsilyl)ethoxycarbonyl-L-leucine allyl ester* *(***14***)* [18]. In a Schlenk-tube equipped with a stirring bar allyl ester **14** (100 mg, 0.199 mmol) was dissolved in dry THF (1 mL) under a nitrogen atmosphere. To the solution were added Pd(PPh_3_)_4_ (23 mg, 20 μmol, 0.1 eq.) and morpholine (173 μL, 1.99 mmol, 10 eq.) and the mixture stirred at room temperature until TLC showed complete consumption of the starting material (2 h). Then the mixture was diluted with EtOAc, transferred to a separatory funnel, washed twice with 1M-NaHSO_4_ soln., once with water and once with brine, the organic layer dried over Na_2_SO_4_ and the solvent evaporated under vacuum. The residual crude Tpseoc-protected amino acid was then coupled with L-leucine *tert-*butyl ester following the general procedure **B** using L-leucine *tert-*butyl ester (56 mg, 0.249 mmol), HBTU (113 mg 0.299 mmol), HOBt (40 mg, 0.299 mmol) and Hünig`s base (104 µL, 0.597 mmol, 3 eq.) yielding Tpseoc-protected dipeptide **33** (113 mg, 0.180 mmol, 90%).

*Methyl ester cleavage in N-2-(triphenylsilyl)ethoxycarbonyl-L-leucine methyl ester* (**15**). In a 25 mL round bottom-flask equipped with a stirring bar methyl ester **15** (100 mg, 0.210 mmol) was dissolved in MeOH/ THF 3:2 (3 mL). Then a 1M-NaOH soln. (630 μL, 0.63 mmol, 3 eq.) was added and the mixture stirred at r.t. until TLC showed complete consumption of the starting material (1 h). The mixture was then diluted with 1M-NaHSO_4_ soln. (25 mL), transferred to a separatory funnel and extracted three times with DCM. The combined organic phases were washed once with water and brine, dried over Na_2_SO_4_ and the solvent evaporated under vacuum. The residual crude Tpseoc-protected amino acid was then coupled with L-leucine *tert-*butyl ester (59 mg, 0.263 mmol) following the general procedure **B** using HBTU (119 mg, 0.315 mmol), HOBt (43 mg, 0.315 mmol) and Hünig`s base (110 µL, 0.630 mmol, 3 eq.) yielding Tpseoc-protected dipeptide **33** (117 mg, 0.185 mmol, 88%).

*Boc-cleavage in N-2-(triphenylsilyl)ethoxycarbonyl-N′-tert-butoxycarbonyl-1,6-diaminohexane* (**21**) [16]. In a Schlenk-tube equipped with a stirring bar diamine **21** (100 mg, 0.183 mmol) was dissolved in dry DCM (1.6 mL) under a nitrogen atmosphere. To the solution were added 1,3-dimethoxybenzene (119 μL, 0.915 mmol, 5 eq.) and TFA (400 μL) and the solution stirred at 0 °C until TLC indicated complete consumption of the starting material (45 min.). The mixture was then evaporated to dryness and the residual crude Tpseoc-protected diamine coupled with *N*-Boc-L-alanine (43 mg, 0.229 mmol) following the general procedure **B** using HBTU (104 mg, 0.274 mmol), HOBt (37 mg, 0.274 mmol) and Hünig`s base (96 µL, 0.630 mmol, 3 eq.) yielding Tpseoc-protected peptide **35** (96 mg, 0.155 mmol, 85%).

*Alloc-cleavage in N-2-(triphenylsilyl)ethoxycarbonyl-N′-allyloxycarbonyl-1,6-diaminohexane* (**22**) [19]. In a Schlenk-tube equipped with a stirring bar diamine **22** (108 mg, 0.203 mmol) was dissolved in dry DCM (2.5 mL) under a nitrogen atmosphere. To the solution were added Pd(PPh_3_)_4_ (12 mg, 10.2 µmol, 0.05 eq.) and Me_2_NH•BH_3_ (24 mg, 0.406 mmol, 2 eq.) and the resulting yellow solution stirred at r.t. until TLC indicated complete consumption of the starting material (2 h). Then methanol (0.5 mL) was added and the mixture stirred for additional 30 min.. Subsequently the solvent was removed under vacuum, the residue taken up in EtOAc and transferred to a separatory funnel. The organic layer was washed twice with sat. NaHCO_3_ soln., once with brine, dried over Na_2_SO_4_ and the solvent removed under vacuum. The residual crude Tpseoc-protected diamine was then coupled with *N*-Boc-L-alanine (48 mg, 0.254 mmol) following general procedure **B** using HBTU (116 mg, 0.305 mmol), HOBt (31 mg, 0.305 mmol) and Hünig`s base (53 µL, 0.305 mmol, 1.5 eq.) yielding Tpseoc-protected peptide **35** (102 mg, 0.165 mmol, 81%).

*Cbz-cleavage in N-2-(triphenylsilyl)ethoxycarbonyl-N′-benzyloxycarbonyl-1,6-diaminohexane* (**23**). In a 25 mL round-bottom flask equipped with gas inlet and a stirring bar diamine **23** (82 mg, 0.141 mmol) was dissolved in dry THF (5 mL). A spatula tip of Pd/C (10%) was added and the mixture placed under an atmosphere of hydrogen and stirred until TLC showed complete consumption of the staring material (8 h). Then the solution was filtered through a pad of Celite and the solvent evaporated under vacuum. The residual crude Tpseoc-protected diamine was then coupled with *N*-Boc-L-alanine (33 mg, 0.176 mmol) following the general procedure **B** using HBTU (80 mg, 0.212 mmol), HOBt (29 mg, 0.212 mmol) and Hünig`s base (37 µL, 0.212 mmol, 1.5 eq.) yielding Tpseoc-protected peptide **35** (74 mg, 0.120 mmol, 85%).

*Phthalimide cleavage in N-2-(triphenylsilyl)ethoxycarbonyl-N′-phthaloyl-1,6-diaminohexane* (**24**) [29]. In a Schlenk-tube equipped with a stirring bar diamine **24** (100 mg, 0.173 mmol) was dissolved in dry toluene (5 mL) under a nitrogen atmosphere. To the solution was added methylhydrazine (182 μL, 3.46 mmol, 20 eq.) and the resulting mixture stirred at 80 °C until TLC indicated complete consumption of the starting material (24 h). Then the solvent was removed under vacuum and the residual crude Tpseoc-protected diamine coupled with *N*-Boc-L-alanine (41 mg, 0.216 mmol) following general procedure **B** using HBTU (99 mg, 0.260 mmol), HOBt (35.1 mg, 0.260 mmol) and Hünig`s base (45 µL, 0.260 mmol, 1.5 eq.) yielding Tpseoc-protected peptide **35** (86 mg, 0.139 mmol, 80%).

*Fmoc-cleavage in N-2-(triphenylsilyl)ethoxycarbonyl-N′-(9-fluorenyl)methoxycarbonyl-1,6-diamino-hexane* (**25**) [1]. In a Schlenk-tube equipped with a stirring bar diamine **25** (150 mg, 0.224 mmol) was dissolved in dry DMF (1 mL) under a nitrogen atmosphere. To the solution was added morpholine (1 mL) and the resulting mixture stirred at r.t. for 1 h. Then the solvent was removed under vacuum and the residual crude Tpseoc-protected diamine coupled with *N*-Boc-L-alanine (53 mg, 0.280 mmol) following the general procedure **B** using HBTU (127 mg, 0.336 mmol), HOBt (45 mg, 0.336 mmol) and Hünig`s base (60 µL, 0.336 mmol, 1.5 eq.) yielding Tpseoc-protected peptide **35** (125 mg, 0.202 mmol, 90%).

### 3.6. Fluoride-ion induced Tpseoc-cleavage in ***27***, ***29***, ***32***, ***33***, ***34*** and ***35***:

*General procedure **C** for the Tpseoc-deprotection of aliphatic primary and secondary amines and alcohols* [8]: In a round bottom flask equipped with gas inlet and a stirring bar the corresponding Tpseoc-protected amine or alcohol was dissolved in dry THF under an atmosphere of nitrogen. To the solution were added 2 mol eq. TBAF•3H_2_O and 2 mol eq. CsF at the temperature noted below and stirred at this temperature until completion of Tpseoc-cleavage was indicated by TLC. Then the mixture was acidified by adding 1M-NaHSO_4_ soln. and stirred another 10 min. at rt. For deprotection of basic amines the solution was then diluted with additional water, transferred to a separatory funnel and extracted three times with EtOAc. The aqueous phase was then made alkaline by addition of 1N-NaOH and again extracted three times with EtOAc. Afterwards the combined organic layers were washed once each with water and brine, dried over Na_2_SO_4_ and the solvent removed in vacuum, leaving the free amine in high purity. An analytical sample could be obtained by silica gel column chromatography with CHCl_3_/MeOH/Et_3_N mixtures as eluent. For non-basic amines and alcohols the mixture was neutralized by addition of sat. NaHCO_3_-soln and extracted three times with EtOAc. The combined organic layers were washed with water and brine and the residual crude product purified by silica gel column chromatography. 

*Deprotection of 3β-[2-(Triphenylsilyl)ethoxycarbonyloxy]androst-5-en-17-one* (**27**). According to general procedure **C** Tpseoc-prasterone **27** (100 mg, 0.162 mmol) was dissolved in dry THF (2 mL) and treated with TBAF•H_2_O (102 mg, 0.323 mmol) and CsF (49 mg, 0.323 mmol) at 0 °C. TLC showed complete cleavage of the Tpseoc-group after 10 min. and chromatographic purification with the eluent mixture light petroleum ether/ethyl acetate 3:2 yielded prasterone (41 mg, 0.142 mmol, 88%) as a white solid. The material proofed to be identical to an authentic sample of prasterone as indicated by NMR-spectra and optical rotation [30].

*Deprotection of N-tert-butoxycarbonyl-1-[2-(triphenylsilyl)ethoxycarbonyl]-L-tryptophan methyl ester (***29***).* According to general procedure **C** Tpseoc-protected thryptophan derivative **29** (100 mg, 0.154 mmol) was dissolved in dry THF (2 mL) and treated with TBAF•3H_2_O (97 mg 0.303 mmol) and CsF (47 mg, 0.303 mmol) at 0 °C. TLC showed complete cleavage of the Tpseoc-group after 10 min. and chromatographic purification with the eluent mixture light petroleum ether/ethyl acetate 3:2 yielded *N*-Boc-tryptophan methyl ester (45 mg, 0.140 mmol, 91%) as colorless crystals. The material proofed to be identical to an authentic sample of *N*-Boc-tryptophan methyl ester as indicated by NMR-spectra and optical rotation [31].

*Deprotection of N-2-(triphenylsilyl)ethoxycarbonyl-L-phenylalanyl-L-leucine tert-butyl ester* (**32**) *to L-phenylalanyl-L-leucine tert-butyl ester* (**36***)*. According to the general procedure **C** Tpseoc-protected dipeptide **32** (100 mg, 0.15 mmol) was dissolved in dry THF (2 mL) and treated with TBAF•3H_2_O (95 mg, 0.30 mmol) and CsF (46 mg, 0.30 mmol) at r.t.. TLC showed complete cleavage of the Tpseoc-group after 6 h and aqueous workup yielded L-phenylalanyl-L-leucine *tert*-butyl ester **36** (46 mg, 0.138 mmol, 92%) as a colorless gum. An analytical sample was obtained by chromatography eluting with CHCl_3_ containing 2.5% MeOH and 1% Et_3_N. The material showed NMR-spectra lacking signals from isomeric dipeptides and optical rotation in agreement with data published before [32] indicating that no racemisation took place during deprotection of the Tpseoc-group. RF: 0.31 (CHCl_3_ +2.5% MeOH). [α]_D_^20^ = −35.4° (c = 1.0, DMF), lit.: -35.2° (c = 1, DMF) [32]. ^1^H-NMR (400 MHz, CDCl_3_): δ 7.66 (d, broad, 1H, *J* = 8.5 Hz, CON**H**), 7.35-7.20 (m, 5H, Phe), 4.50 (dt, 1H, *J* = 8.7 Hz/5 Hz, α-C**H**-Leu), 3.65 (dd, 1H, *J* = 9.2 Hz/4 Hz, α-C**H**-Phe), 3.24 (dd, 1H, *J* = 13.7 Hz/4 Hz, β-C**H**-Phe), 2.73 (dd, 1H, *J* = 13.7 Hz/ 9.2 Hz, β-C**H**-Phe) 1.66-1.43 (m, 5H, β/γ-C**H**-Leu, N**H**_2_), 1.46 (s, 9H, *t-*Bu), 0.97-0.92 (m, 6H, δ-C**H**_3_-Leu). ^13^C-NMR (100.6 MHz, CDCl_3_): δ 174.0, 172.3, 137.8, 129.8, 128.8, 126.9, 81.8 (*t-*Bu), 56.4, 51.0, 41.9, 40.9, 28.1 (*t-*Bu), 25.0, 23.0, 22.1.

*Deprotection of N-2-(triphenylsilyl)ethoxycarbonyl-L-leucyl-L-leucine tert-butyl ester* (**33**) *to L-leucyl-L-leucine tert-butyl ester* (**37**). According to the general procedure **C** Tpseoc-protected dipeptide **33** (179 mg, 0.284 mmol) was dissolved in dry THF (3 mL) and treated with TBAF•3H_2_O (179 mg, 0.568 mmol) and CsF (86 mg, 0.568 mmol) at r.t.. TLC showed complete cleavage of the Tpseoc-group after 6 h and aqueous workup yielded L-leucyl-L-leucine *tert*-butyl ester **37** (81 mg, 0.270 mmol, 95%) as a colorless gum. An analytical sample was obtained by chromatographic purification eluting with CHCl_3_ containing 1% MeOH and 1% Et_3_N. The material showed NMR-spectra lacking signals from isomeric dipeptides. [α]_D_^20^ = −21.5° (c = 1.0, CHCl_3_). FAB-MS calcd for C_16_H_32_N_2_O_3_: m/z 301.3 [M+H]^+^, 245.2 [M-*t*Bu+H]^+^. FT-ICR-MS: m/z [M+Na]^+^ calcd for C_16_H_32_N_2_O_3_: 301,2486 found: 301.2486. ^1^H-NMR (400 MHz, CDCl_3_): δ 7.58 (d, broad, 1H, *J* = 8.5 Hz, CON**H**), 4.46 (dt, 1H, *J* = 8.7 Hz/5.1 Hz, α-C**H**-Leu-ester), 3.40 (dd, 1H, *J* = 8.2 Hz/3.6 Hz, α-C**H**-Leu), 1.76-1.22 (m, 8H, β/γ-C**H**-Leu, N**H**_2_), 1.44 (s, 9H, *t-*Bu_H_), 0.97-0.89 (m, 12H, δ-C**H**_3_). ^13^C-NMR (100.6 MHz, CDCl_3_): δ 175.5, 172.6, 81.8 (*t-*Bu_H_), 53.8, 51.1, 44.3, 42.0, 28.2 (*t-*Bu), 25.2, 25.1, 23.6, 23.1, 22.3, 21.6.

*Deprotection of N-2-(triphenylsilyl)ethoxycarbonyl-L-prolyl-L-leucine tert-butyl ester* (**34**) *to L-prolyl-L-leucine tert-butyl ester (***38***)* [33,34]. According to the general procedure **C** Tpseoc-dipeptide **34** (112 mg, 0.182 mmol) was dissolved in dry THF (2 mL) and treated with TBAF•3H_2_O (115 mg, 0.364 mmol) and CsF (55 mg, 0.364 mmol) at rt. TLC showed complete cleavage of the Tpseoc-group after 90 min. and aqueous workup yielded L-prolyl-L-leucine *tert*-butyl ester **38** (45 mg, 0.162 mmol, 89%) as colorless crystals. An analytical sample was obtained by chromatographic purification eluting with CHCl_3_ containing 1% MeOH and 1% Et_3_N. The material showed NMR-spectra lacking signals from isomeric dipeptides. [α]_D_^20^ = −14.6° (c = 1.0, CHCl_3_). FAB-MS calcd for C_15_H_28_N_2_O_3_: m/z 285.2 [M+H]^+^, 229.2 [M-*t*Bu+H]^+^. ^1^H-NMR (400 MHz, CDCl_3_): δ 7.93 (d, broad, 1H, *J* = 8.6 Hz, CON**H**), 4.40 (dt, 1H, *J* = 8.8 Hz/5 Hz, α-C**H**-leu), 3.74 (dd, 1H, *J* = 9.2 Hz/5.3 Hz, α-C**H**-Pro), 3.0 (dt, 1H, *J* = 10.0 Hz/6.9 Hz, δ-C**H**_2_-Pro), 2.90 (dt, 1H, *J* = 10.2 Hz/6.2 Hz, δ-C**H**_2_-Pro), 2.43 (s, broad, 1H, NH), 2.18-2.06 (m, 1H, β-C**H**-Pro), 1.92-1.82 (m, 1H, β-C**H**-Pro), 1.73-1.47 (m, 5H, β/γ-C**H**-Leu, γ-C**H**-Pro), 1.44 (s, 9H, *t-*Bu), 0.96-0.87 (m, 6H, δ-C**H**-Leu). ^13^C-NMR (100.6 MHz, CDCl_3_): δ 174.9, 172.5, 81.7 (*t-*Bu), 60.6, 50.9, 47.4, 41.8, 31.0, 28.1 (*t-*Bu), 26.3, 25.2, 23.0, 22.2.

*Deprotection of N-2-(triphenylsilyl)ethoxycarbonyl-N′-(N-tert-butoxycarbonyl)-L-alanyl-1,6-diamino-hexane* (**35**) *to N-(N′-tert-butoxycarbonyl-L-alanyl)-1,6-diaminohexane* (**39**): According to general procedure **C** Tpseoc-peptide **35** (125 mg 0.202 mmol) was dissolved in dry THF (2 mL) and treated with TBAF•3H_2_O (128 mg, 0.404 mmol) and CsF (61 mg, 0.404 mmol) at r.t.. TLC showed complete cleavage of the Tpseoc-group after 24 h and aqueous workup yielded *N*-(*N′*-Boc-L-alanyl)-1,6-diaminohexane **39** (53 mg, 0.202 mmol, 91%) as a colorless gum. An analytical sample was obtained by chromatography with the eluent mixture chloroform/methanol 9:1-8:2 containing 1% Et_3_N. [α]_D_^20^ = −25.5° (c = 1.0, CHCl_3_). FAB-MS calcd for C_14_H_29_N_2_O_3_: m/z 288.2 [M+H]^+^, 188.2 [M-BOC+H]^+^. FT-ICR-MS: m/z [M+Na]^+^ calcd for C_14_H_29_N_3_O_3_: 288,2282 found: 288,2284. ^1^H-NMR (400 MHz, CDCl_3_): δ 6.69 (s, broad, 1H, -CON**H**), 5.37 (d, broad, 1H, *J* = 7.1 Hz, OCON**H**), 4.19-4.07 (m, 1H, α-C**H**), 3.97-3.82 (m, 2H, C**H**_2_NHCO), 3.25-3.15 (m, 2H, chain-C**H**_2_), 2.75-2.66 (m, 2H, chain-C**H**_2_), 1.53-1.43 (m, 4H, chain-C**H**_2_), 1.42 (s, 9H, *t-*Bu), 1.35-1.27 (m, 7H, chain-C**H**_2_/β-C**H**_3_). ^13^C-NMR (100.6 MHz, CDCl_3_): δ 172.9, 155.8, 80.0 (*t-*Bu_H_), 51.1 (α-**C**H), 41.2, 39.4, 31.8, 29.4, 28.4 (*t-*Bu), 26.5, 26.3, 18.7 (β-**C**H_3_).

## 4. Conclusions

In summary a new fluoride ion cleavable amino/alcohol protecting group based on the 2-(triphenyl-silyl)ethoxycarbonyl- (“Tpseoc”-) moiety was developed and installed into a series of amino acids and peptides and a steroid alcohol. The protected derivatives were synthesized via a short and efficient route starting from commercially available triphenylvinylsilane. Contrary to the Teoc-group, the Tpseoc-group proved to be highly resistant to acidic conditions necessary to cleave *tert-*butyl esters and the Boc-group. The Tpseoc-group was found to be compatible with a wide range of conditions, e.g., basic conditions needed to cleave methyl-esters and the Fmoc-group, catalytic hydrogenation with Pd/C in various solvents, treatment with Pd-catalysts in presence of an allyl-scavenger as applied in cleavage of allyl-based protecting groups and methylhydrazine, used in deprotection of phthaloyl-groups. Cleavage of the Tpseoc-group was achieved by treatment with TBAF•3H_2_O and CsF with cleavage times ranging from 10 minutes to 24 hours. The observed cleavage kinetics are significantly enhanced compared to those reported for of Teoc- and SES-group [1]. Its general applicability as carbamate or carbonate protecting group and the fact that it can be introduced via standard methods employing a stable crystalline chloroformate reagent together with its UV-detectability make the Tpseoc-group a promising candidate for being adopted into the pool of protecting groups used more frequently in organic synthesis. Especially the very fast cleavage from electron poor amines and alcohols and its orthogonality to acid and fluoride labile linkers should make the Tpseoc-group an interesting choice as protecting group in automated solid-phase synthesis of oligonucleotides, peptides and saccharides. Adjustment of the Tpseoc-groups reactivity pattern could be established by introduction of electron releasing or electron withdrawing residues on the phenyl groups of the triphenylsilyl-moiety.
